# Detection, characterization, and evolutionary inferences regarding a novel virus identified in *Amblyomma dissimile* Koch, 1844 (Acari: Ixodidae) from the Amazon, Pará, Brazil

**DOI:** 10.1007/s00705-026-06764-5

**Published:** 2026-10-23

**Authors:** Lucas Henrique da Silva e Silva, Sandro Patroca da Silva, Paulo Henrique Gomes de Castro, Klebson Demelas Maurício, Fabio Silva da Silva, Daniel Damous Dias, Leonardo Henrique Almeida Hernández, Sâmia Luzia Sena da Silva, Hanna Carolina Farias Reis, Lorena Evelyn Cordovil dos Santos, Bruna Laís Sena do Nascimento, Roberto Carlos Feitosa Brandão, Durval Bertram Rodrigues Vieira, Bruno Tardelli Diniz Nunes, Ana Cecília Ribeiro Cruz, Joaquim Pinto Nunes Neto

**Affiliations:** 1 Post-graduate program in Parasitary Biology in the Amazon, Center of Biological and Health Sciences, State of Pará University, Belém, 66095-662 Brazil; 2 Department of Arbovirology and Hemorrhagic Fevers, Evandro Chagas Institute - IEC/SVS/MS, Ananindeua, 67030-000 Brazil; 3 National Primate Center – CENP, Ecology and Environment Section (SEEMA), Ananindeua, 67030-000 Brazil

## Abstract

Ticks are among the primary vectors of infectious agents of medical and veterinary importance, second only to mosquitoes in the number of pathogens transmitted to humans. These arthropods are capable of transmitting a wide diversity of microorganisms, including protozoa, bacteria, and viruses. Notable among tick-borne viruses are those associated with viral encephalitis, which cause severe acute and chronic neurological infections in humans. This study aimed to characterize the RNA virome of an *Amblyomma dissimile* specimen collected from a *Boa constrictor* rescued in the municipality of Ananindeua, Pará state, in the Brazilian Amazon, using an RNA metagenomic sequencing approach. A total of eleven viral sequences were identified, distributed across five families: *Chuviridae* (*n* = 2), *Rhabdoviridae* (*n* = 2), *Orthomyxoviridae* (*n* = 5), *Nodaviridae* (*n* = 1), and *Orthototiviridae* (*n* = 1). A key finding was the identification of *Dissimile tick rhabdovirus* a potential new member of the *Rhabdoviridae* family described here for the first time, with no prior records in the scientific literature. Additionally, segments of *Granville quaranjavirus* were detected in Brazil for the first time, expanding the known geographic distribution of this virus. These results highlight the underrepresentation of viral diversity associated with Brazilian ticks in reference databases, underscoring the need to expand metagenomic surveillance and virological characterization efforts in the country, particularly given the potential impacts of climate change on tick distribution and virus circulation dynamics.

**Supplementary Information:**

The online version contains supplementary material available at https://doi.org/10.1007/s00705-026-06764-5.

## Introduction

Ticks are among the primary vectors of infectious agents of medical and veterinary importance, surpassed only by mosquitoes in the number of pathogens transmitted to humans [25]. These arthropods are capable of transmitting a wide diversity of microorganisms, including protozoa, bacteria, and viruses [5, 57, 62, 88, 89]. Among tick-borne viruses, those associated with viral encephalitis are particularly notable, as they cause severe acute and chronic neurological infections in humans [99]. Recently, a review conducted by Silva et al., [93] compiled the major tick-borne viruses of public health relevance, including agents associated with severe clinical conditions and fatal cases. The study also highlighted the increasing discovery, through metagenomic approaches, of numerous unclassified viruses in ticks of the families Ixodidae and Argasidae, demonstrating that the viral diversity of these ectoparasites remains largely underexplored.

The family Ixodidae comprises four genera of major relevance in the transmission of pathogens of medical and veterinary importance: *Ixodes*, *Amblyomma*, *Rhipicephalus*, and *Dermacentor* [4]. Among them, the genus *Amblyomma* Koch, 1844 stands out for its high diversity, comprising approximately 139 species widely distributed across the Indomalayan, Afrotropical, Australian, Nearctic, and Neotropical regions, with the latter representing its primary center of diversity [19]. *Amblyomma dissimile* Koch, 1844 is a species widely distributed throughout the Americas [18, 35] and is frequently found parasitizing amphibians and reptiles, including iguanas, snakes, lizards, and chelonians [79]. Occasional records also describe its occurrence on atypical hosts, such as dogs, cattle, and humans [18, 27, 33]. Various agents have been described in association with this species, including unclassified viruses belonging to the families *Chuviridae* and *Orthomyxoviridae*, the latter including a potential new member of the genus *Quaranjavirus* [85].

Metagenomic studies conducted on ticks of the genus *Amblyomma* have revealed significant diversity among viruses belonging to families of public and veterinary health importance, as well as a large number of unclassified viruses [52] (Ni et al., 2023) [59]. These findings highlight that the viral diversity associated with these ectoparasites remains largely underestimated. In this context, climate change and its effects on the geographic distribution, abundance, and population dynamics of ticks may alter the circulation and emergence patterns of viruses transmitted by these arthropods [67]. Concurrently, the limited number of studies on the virome of tick populations in developing countries restricts knowledge of regional viral diversity and may contribute to the underreporting of tick-borne diseases in South America [17, 55, 81, 97, 107]. Thus, investigating the tick virome is essential for understanding the mechanisms of virus maintenance and circulation in natural environments, identifying emerging viral lineages, and supporting surveillance strategies capable of anticipating future outbreaks of diseases associated with these vectors [11, 12, 77, 81, 92].

In Brazil, studies characterizing viral diversity in *Amblyomma* ticks remain scarce, particularly in the Brazilian Amazon, highlighting a significant gap in knowledge regarding the virosphere associated with these ectoparasites and their potential role in virus maintenance and circulation in the region. Among the few studies conducted in the country, notable reports include the detection of medically important viruses in ticks of this genus, such as members of the *Orthobunyavirus* genus, e.g., viruses belonging to the Bunyamwera complex [7]. Additionally, experimental studies have demonstrated that *Amblyomma* species are capable of acquiring and maintaining orthoflaviviruses of public health significance, suggesting that these ticks may play a role in maintaining these viruses in nature and potentially influence their epidemiological dynamics in South America [26].

Although *Amblyomma dissimile* is primarily associated with reptiles and amphibians, its occurrence across diverse vertebrate hosts highlights the ecological complexity of tick–host interactions [6, 79]. Investigating the virome of wildlife-associated ticks can provide valuable baseline information on viral diversity and may contribute to the early recognition of viruses with potential for host switching and spillover [8, 30]. This is particularly relevant under ongoing environmental and climatic changes, which can alter tick distribution, abundance, phenology, and host availability, potentially reshaping tick–virus–host interactions [32, 69]. Therefore, studying tick species that are not traditionally regarded as medically or veterinary important can complement surveillance of established vector species and contribute to a broader understanding of viral diversity and emergence at wildlife interfaces.

The Amazon rainforest is recognized as one of the world’s largest reservoirs of viral diversity [54, 100]. Its climatic and geographic characteristics, marked by high year-round temperatures, high humidity, intense rainfall, and extensive forest cover, favor the maintenance and proliferation of hematophagous arthropods such as mosquitoes, sand flies, and ticks. This makes the biome a strategic environment for entomovirological surveillance and the detection of emerging viruses [66, 76, 100]. Despite this, most research conducted in the Amazon has focused on mosquito-borne arboviruses [3, 76], while the viral diversity associated with ticks, particularly species of the genus *Amblyomma*, remains underexplored. Consequently, knowledge regarding novel viruses and viral variants potentially relevant to vertebrate health remains limited. Given this context, and considering the high biodiversity of the Amazon and its potential for the emergence and re-emergence of viruses of public health importance, this study aimed to characterize RNA viruses from an *Amblyomma dissimile* specimen collected from a *Boa constrictor* rescued in the municipality of Ananindeua, Pará state, in the Brazilian Amazon, using an RNA metagenomic sequencing approach.

## Materials and methods

### Study area

**Fig. 1:**
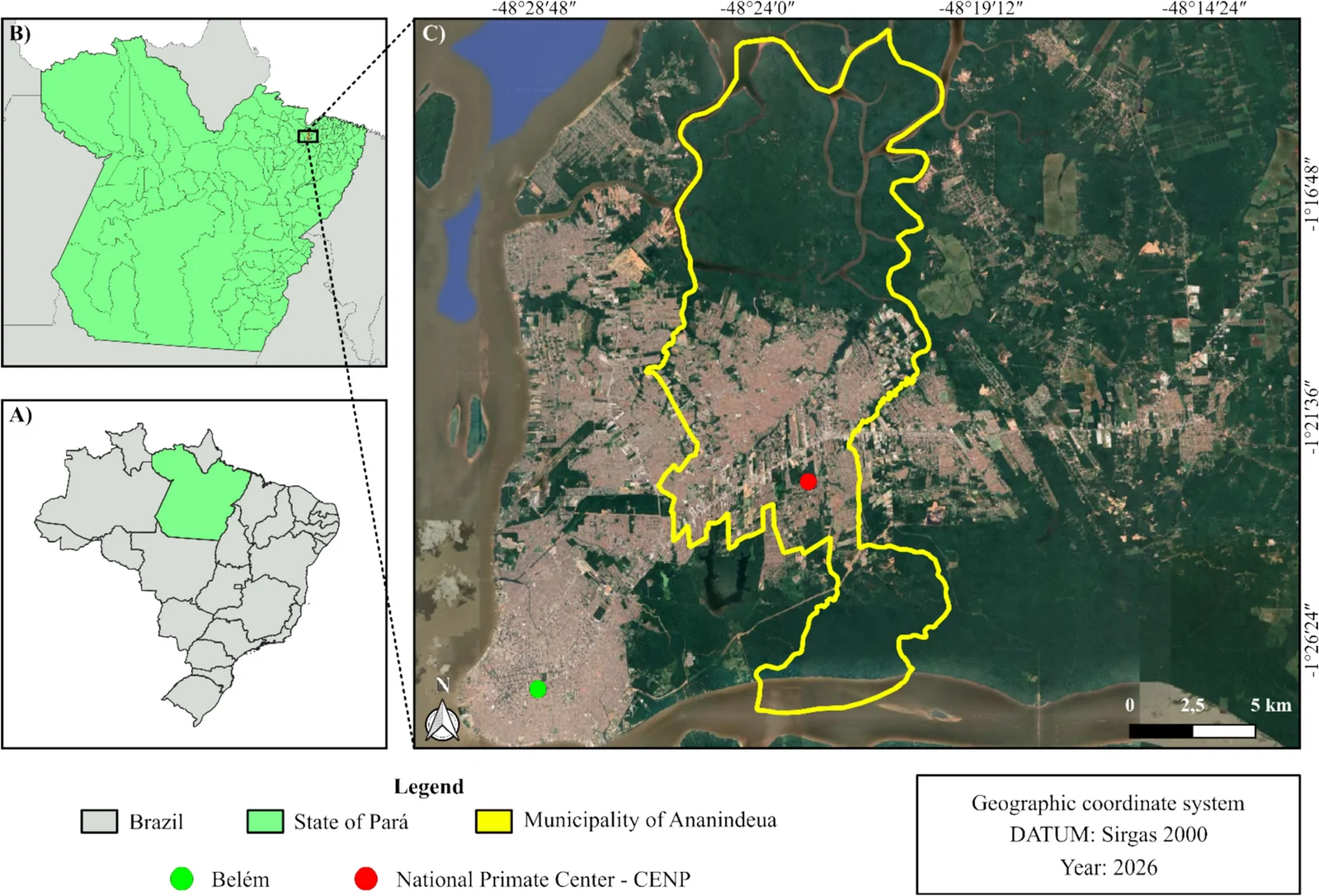
Location of the city of sample origin. **A**) Location of the state of Pará in Brazil. **B**) Location of the municipality of Ananindeua in the state of Pará. **C**) Territorial boundary of the municipality of Ananindeua (yellow line) overlaid on a satellite image, with the collection point at the National Primate Center (CENP) indicated by the red circle. The figure was created using QGIS v. 3.44.11 (https://qgis.org/en/site/, accessed on July 24, 2026) 72with cartographic data from the Brazilian Institute of Geography and Statistics [40] (https://www.ibge.gov.br/, accessed on July 24, 2026)

The *A. dissimile* specimen was collected from a *Boa constrictor* rescued near the National Primate Center (CENP) in Ananindeua, Pará (PA) (Fig. 1). The animal was sent to the CENP’s Ecology and Environment Section (SAEMA) for assessment, species identification, and ectoparasite removal. The tick was removed using forceps, placed in a Falcon tube, and donated to the Medical Entomology Laboratory of the Arbovirology and Hemorrhagic Fevers Section (SEARB) at the Evandro Chagas Institute (IEC). The sample was stored in a −80 °C freezer for preservation and subsequent taxonomic identification.

### Taxonomic identification

#### Morphological and molecular identification

Morphological taxonomic identification was performed at the Medical Entomology Laboratory of SEARB/IEC. Specimens were identified by observing morphological characteristics using a stereomicroscope on a refrigerated stage (−28 °C), based on the dichotomous key proposed by Guimarães et al., [34]. Subsequently, the specimen was assigned an internal alphanumeric code starting with “AR” (for ARthropod), followed by a six-digit number; the sample was then stored in a −80 °C freezer for preservation. The molecular identification of the ticks had been previously validated using mitochondrial molecular markers, including the *COXI* gene and the 13 protein-coding regions (GenBank accession: PZ569664). The present study uses the same sample for virus investigation.

### Molecular biology

#### Pre-extraction of total RNA

The tick specimen intended for the investigation of RNA viruses by high-throughput sequencing was subjected to surface decontamination prior to total RNA extraction, with the aim of reducing the presence of external contaminants. Initially, the specimen was washed for 30 s in 1 mL of 70% ethanol, followed by a 30-second wash in 1 mL of phosphate-buffered saline (PBS; pH 7.4). Subsequently, the specimen was washed with nuclease-free water (Qiagen) and vortexed. After the decontamination procedure, the arthropod was carefully removed from the solution and transferred to a sterile 2-mL microcentrifuge tube, where it was kept until the subsequent total RNA extraction step.

#### Total RNA extraction

A single tick specimen was selected and homogenized using a TissueLyser II (Qiagen, Hilden, Germany) with 500 µL of Dulbecco’s phosphate-buffered saline (PBS) containing 2% penicillin and streptomycin, 1% amphotericin B, 5% fetal bovine serum, and a sterile 5 mm-diameter tungsten bead, at a frequency of 25 Hz for 2 min. Subsequently, the material was centrifuged at 12,000 × g and 4 °C for 10 min. Following this step, 140 µL of the supernatant was collected for RNA extraction and purification using the QIAamp^®^ Viral RNA Mini Kit (Qiagen). After purification, the RNA was quantified using the Qubit RNA HS Assay Kit (Invitrogen, Waltham, MA, USA) on a Qubit 4.0 instrument (Invitrogen), in accordance with the manufacturer’s recommendations.

#### Double-stranded cDNA synthesis and genomic library preparation

cDNA preparation from RNA involved the synthesis of the first and second cDNA strands using the SuperScript™ IV VILO™ Master Mix (Invitrogen) and Second Strand cDNA Synthesis Kit (Invitrogen), respectively. cDNA purification was performed using the PureLink^®^ PCR Purification Kit (Invitrogen). All steps followed the respective kit manufacturers’ recommendations. The synthesized cDNA was quantified using the Qubit™ dsDNA HS Assay Kit (Invitrogen) on a Qubit 4.0 instrument. The genomic library was prepared according to the Nextera XT DNA Kit (Illumina, San Diego, CA, USA) guidelines; the library was then quantified using the same process applied to the cDNA, and the generated fragment sizes were assessed using a 2100 Bioanalyzer instrument (Agilent Technologies, Santa Clara, CA, USA) with the Bioanalyzer High Sensitivity DNA Analysis Kit (Agilent Technologies). Following these quality checks, the library was sequenced on the NextSeq 500 platform (Illumina, Inc.) using the NextSeq 500/550 High Output Kit v2.5 (300 Cycles) with a paired-end approach, in accordance with the manufacturer’s recommendations.

### Bioinformatics

#### Quality control

The obtained sequencing data were initially subjected to quality assessment using Fastp v.0.23.4 (https://github.com/OpenGene/fastp) [16], configured to remove adapter sequences and reads with Phred base quality < 20 and lengths shorter than 50 nt. Ribosomal reads were removed using SortMeRNA v. 2.1 [44] based on default parameters and the integrated default database (smr_v4.3_default_db.fasta), derived from the following reference sources: SILVA 138 SSURef NR99 (for 16 S and 18 S rRNAs), SILVA 132 LSURef (for 23 S and 28 S rRNAs), and Rfam v14.1 (for 5 S and 5.8 S rRNAs).

#### Genome assembly and viral classification

Reads were assembled *de novo* using MEGAHIT v.1.2.9 (https://github.com/voutcn/megahit) [50] with default settings (k-mers of 21, 29, 39, 59, 79, 99, 119, and 141 nt). Contigs were first screened using DIAMOND v. 2.1.9 (BlastX mode) against the NCBI nr and RefSeq databases. Top hits were then confirmed via BlastX (https://blast.ncbi.nlm.nih.gov) against the same databases using default parameters and visualized using MEGAN v. 6.25.10 [38].

#### Functional domain annotation

Viral contigs previously examined using MEGAN 6.25.10 were exported to Geneious Prime 2025.1.3 [43], where inspections and Open Reading Frame (ORF) investigations were conducted. The identified informative genes were extracted and submitted for functional domain annotation using the InterPro online tool (https://www.ebi.ac.uk/interpro/search/sequence/) [70]. The output files were imported into Geneious Prime v.2025.1.3 for visualization and editing. Additional figure editing was performed using Inkscape v. 1.4.2 (https://inkscape.org/release/inkscape-1.4.2/windows/64-bit/msi/dl/).

#### Inspection and phylogenetic analysis

Following inspection of the contigs, and based on criteria defined by the International Committee on Taxonomy of Viruses (ICTV; https://ictv.global), informative genes were extracted and aligned with ICTV-provided databases for each family under investigation, as well as with closely related sequences identified via BlastX in the GenBank (NCBI) database. Subsequently, using IQTREE v.3.0.1 software [106], the best-fit substitution model was determined according to the Akaike Information Criterion (AIC); this was followed by an assessment of the phylogenetic signal and phylogenetic reconstruction using Maximum Likelihood, with support values calculated based on 1,000 replicates and branch support estimated using ultrafast bootstrap (UFB). The final consensus tree was visualized and annotated using FigTree v.1.4.4 [74]. Final illustrations were produced using Inkscape v.1.3.2 (https://inkscape.org/) 39.

## Results

*Amblyomma dissimile* specimen was confirmed based on the *COXI* molecular marker, as well as through the 13 protein-coding genes (PCGs). The arthropod sample was suitable for high-throughput sequencing, generating a total of approximately 38 million reads, with about 33 million reads validated for genome assembly. Of the eligible reads, a total of 12.6% were classified as viral reads (Table 1).

**Table 1:** General information about the samples and information post-sequencing and quality control The reads were assembled and submitted to BLASTx. A total of eleven viral sequences were identified, distributed among five families: Chuviridae (*n* = 2), Rhabdoviridae (*n* = 2), Orthomyxoviridae (*n* = 5), Nodaviridae (*n* = 1), and Orthototiviridae (*n* = 1) (Table 2)

Specie (GenBank acess)	Samples	Stage of tick	Physiological state	Collection municipality
*A. dissimile* (PZ569664)	AR899864	Adult ♂ (*n* = 1)	Semi-engorged	Ananindeua
	**Total reads**	**Fastp**	**SortMeRNA**	**Viral**
*A. dissimile* (PZ569664)	37,326,920	32,050,066	27,359,152	12.6%

### Viral Families

#### Chuviridae

**Fig. 2:**
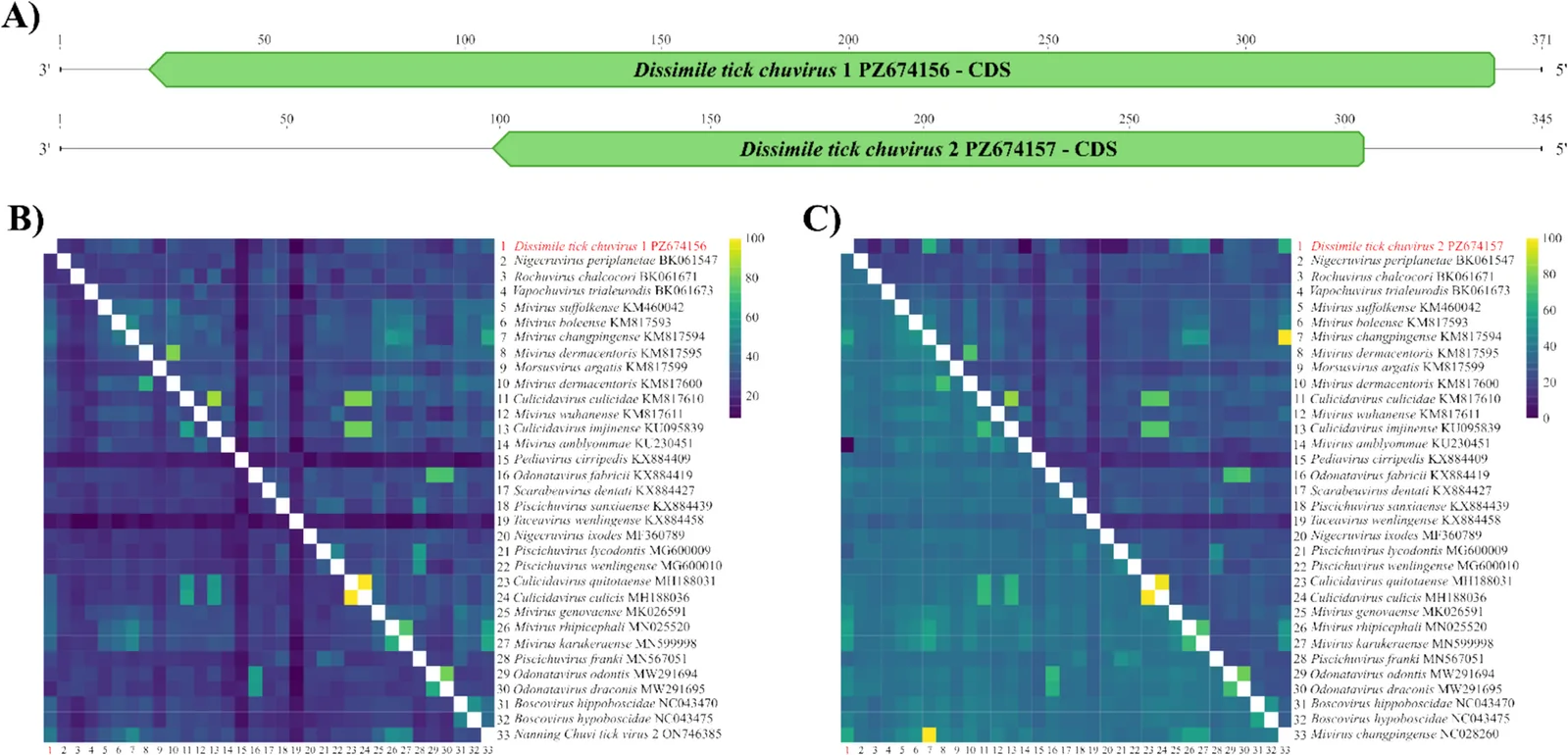
Organization of the identified genes. **A**) Comparison between the coding sequences (CDSs) of *Dissimile tick chuvirus* 1 (GenBank: PZ674156) and *Dissimile tick chuvirus* 2 (GenBank: PZ674157). **B**) Pairwise identity matrix heatmaps for the sequences used in the analysis. **C**) Pairwise identity matrix heatmaps for the sequences used in the analysis. The lower and upper triangles show nucleotide and amino acid identity percentages, respectively. The sequences obtained are highlighted in red

Two sequences related to the family Chuviridae were detected. Blastx results linked *Dissimile tick chuvirus* 1 PZ674156 (DTCV1) and *Dissimile tick chuvirus* 2 PZ674157 (DTCV2) to the nucleoproteins of *Nanning Chuvi tick virus* 2 (ON746385) and *Mivirus changpingense* (NC028260), respectively (Table 2). Although associated with the respective viruses, the CDS regions did not exhibit domain signatures (Fig. 2A). Upon evaluation of nucleotide and amino acid identity, the detected sequences showed values highly divergent from the nucleoproteins of representatives of the family *Chuviridae* (Fig. 2B, C).

**Table 2:** Viruses identified in specimens of the genus *Amblyomma* by high-throughput sequencing, including taxonomic classification, the viral segment or gene detected, and sequence identity relative to reference viruses. QC = Query cover; NP = Nucleoprotein; P = Polymerase; RdRp = RNA-dependent RNA polymerase

Tick species	Molecule type	Virus name (GenBank)	Classification	Closest virus (GenBank Accession)	*Blastx*			
					Region	Identity (%)	QC (%)	E-value
*Amblyomma dissimile*	-ssRNA	*Granville quaranjavirus* (PZ674163)	*Orthomyxoviridae*	*Granville quaranjavirus* (MZ502305.1)	PB1	98.29	91	0.0
		*Granville quaranjavirus* (PZ674164)	*Orthomyxoviridae*	*Granville quaranjavirus* (MZ502308.1)	PB2	98.38	68	0.0
		*Granville quaranjavirus* (PZ674162)	*Orthomyxoviridae*	*Granville quaranjavirus* (MZ502307.1)	PA	96.89	88	0.0
		*Granville quaranjavirus* (PZ674160)	*Orthomyxoviridae*	*Granville quaranjavirus* (MZ502306.1)	HA	97.51	90	0.0
		*Granville quaranjavirus* (PZ674161)	*Orthomyxoviridae*	*Granville quaranjavirus* (MZ502304.1)	NP	97.6	70	0.0
		*Dissimile tick chuvirus 1* (PZ674156)	*Chuviridae*	*Nanning Chuvi tick virus* 2 (ON746385)	NP	43.30	97	4e^− 18^
		*Dissimile tick chuvirus 2* (PZ674157)	*Chuviridae*	*Mivirus changpingense* (NC028260)	NP	68.97	50	1e^− 16^
	+ssRNA	*Dissimile tick rhabdovirus* (PZ674158)	*Rhabdoviridae*	*Alpharicinrhavirus blanchseco* (NC076415)	P	84.90	56	0.0
		*Dissimile tick rhabdovirus* (PZ674159)	*Rhabdoviridae*	*Alpharicinrhavirus blanchseco* (NC076415)	NP	77.65	97	0.0
		*Lone star tick nodavirus* (PZ674165)	*Nodaviridae*	*Lone star tick nodavirus* (KX774634)	P	98.85	100	0.0
	dsRNA	*Orthototi-like virus* (PZ674166)	*Orthototiviridae*	*Orthototiviridae* sp. (ON812683)	RdRp	90	99	4e^− 34^

**Fig. 3:**
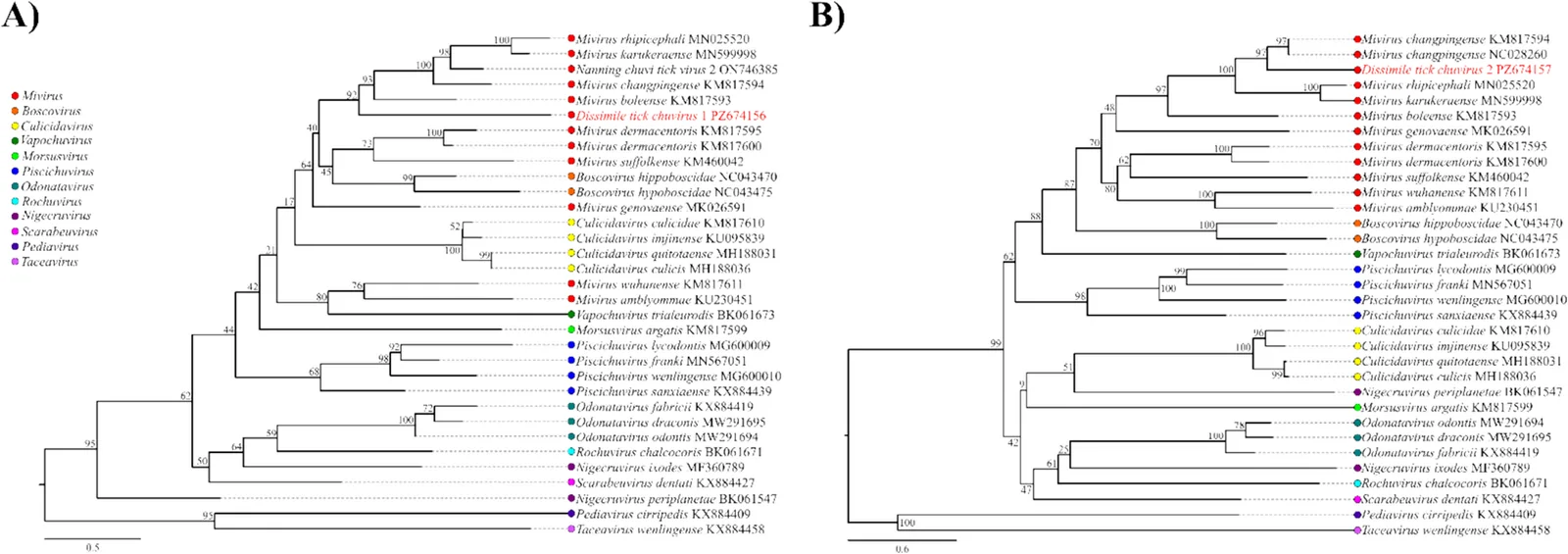
Detected sequences of viruses related to the family *Chuviridae* and their phylogenetic relationships. **A**) Phylogenetic tree constructed using the LG + G4 amino acid substitution model for the CDS. **B**) Phylogenetic tree constructed using the LG + F+G4 amino acid substitution model for the CDS. Sequences obtained in this study are highlighted in red

Upon performing nucleotide and amino acid sequence alignments with representatives of the *Chuviridae* family, the sequences were observed to be highly divergent (Supplementary S1). Phylogenetic inferences revealed that DTCV1 and DTCV2 cluster within clades composed of representatives of the genus *Mivirus*, supported by high statistical values (UFB > 92%), thereby corroborating the evolutionary affinity of these viruses with other members of the genus (Fig. 3).

#### Rhabdoviridae

**Fig. 4:**
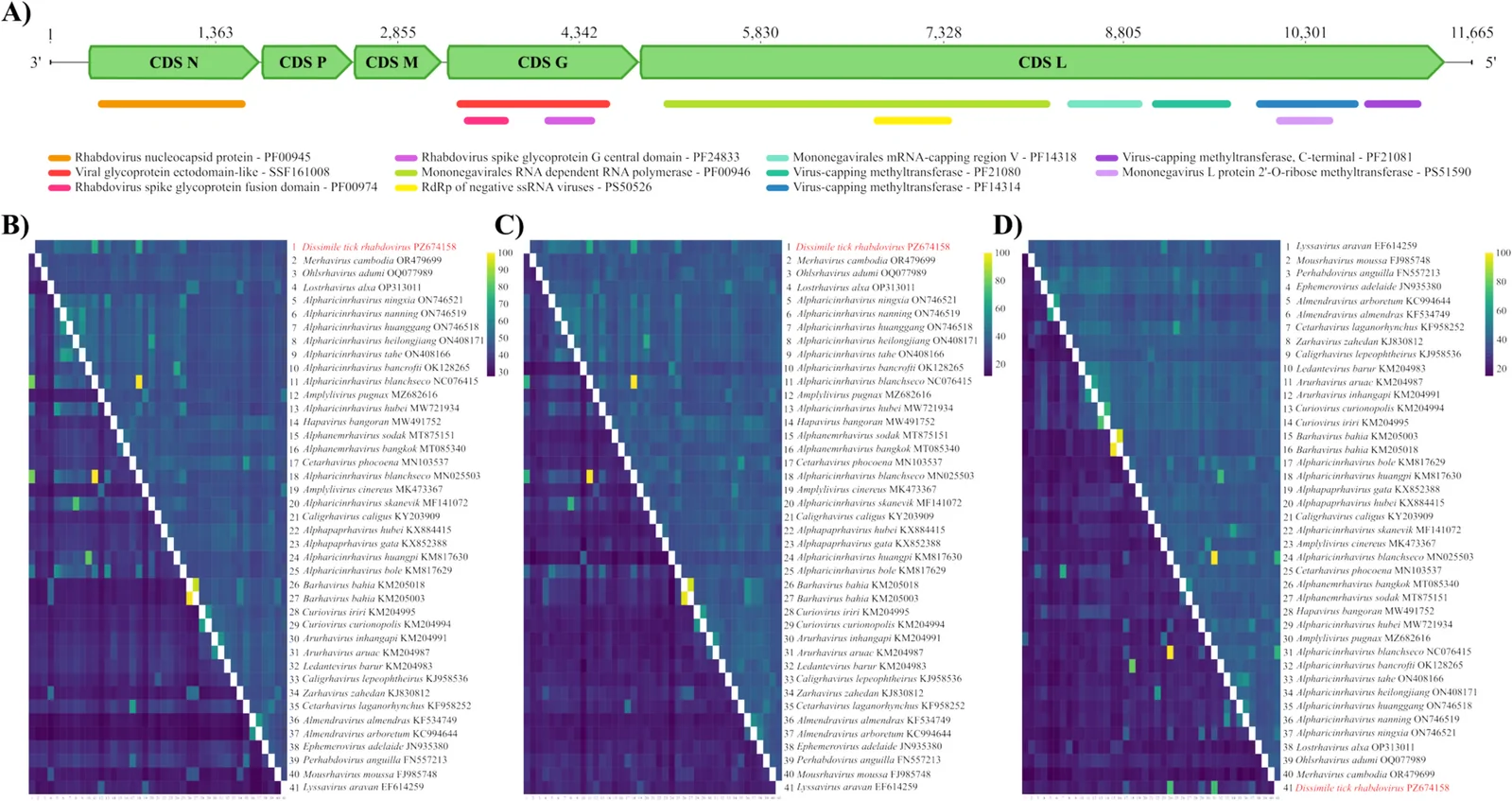
Genomic characterization of *Dissimile tick rhabdovirus* PZ674158. **A**) Genomic organization of *Dissimile tick rhabdovirus* genes, showing five coding regions (CDS N, CDS P, CDS M, CDS G, and CDS L) and the location of identified domains. Each domain is represented by a specific color. **B**) Heatmap of amino acid and nucleotide identity for the *L* gene based on pairwise comparisons among representative sequences of the *Rhabdoviridae* family; **C**) Heatmap of amino acid and nucleotide identity for the *G* gene; **D**) Heatmap of amino acid and nucleotide identity for the *N* gene; the color scale indicates the percentage of identity. The lower and upper triangles show amino acid and nucleotide identity percentages, respectively. The obtained sequence is highlighted in red. L = Polymerase; G = Glycoprotein; NP = Nucleoprotein; P = Phosphoprotein; M = Matrix protein

A virus with all ORFs intact and a total length of 11,665 bp was detected, exhibiting five gene regions designated as *L*, *G*, *M*, *P*, and *N*. The identified genes encode the polymerase, glycoprotein, matrix protein, phosphoprotein, and nucleoprotein, respectively. Furthermore, conserved functional domains were identified within the *L*, *G*, and *N* genes (Fig. 4A).

Data generated by Blastx link the obtained sequence to *Alpharicinrhavirus blanchseco* (NC076415) with 84.90% identity, considering the complete genome (Table 2). Amino acid and nucleotide alignments of the *L* gene yielded 84.5% and 72.6% identity, respectively, with the *Alpharicinrhavirus blanchseco* (NC076415) sequence (Fig. 4B; Supplementary S2).

**Fig. 5:**
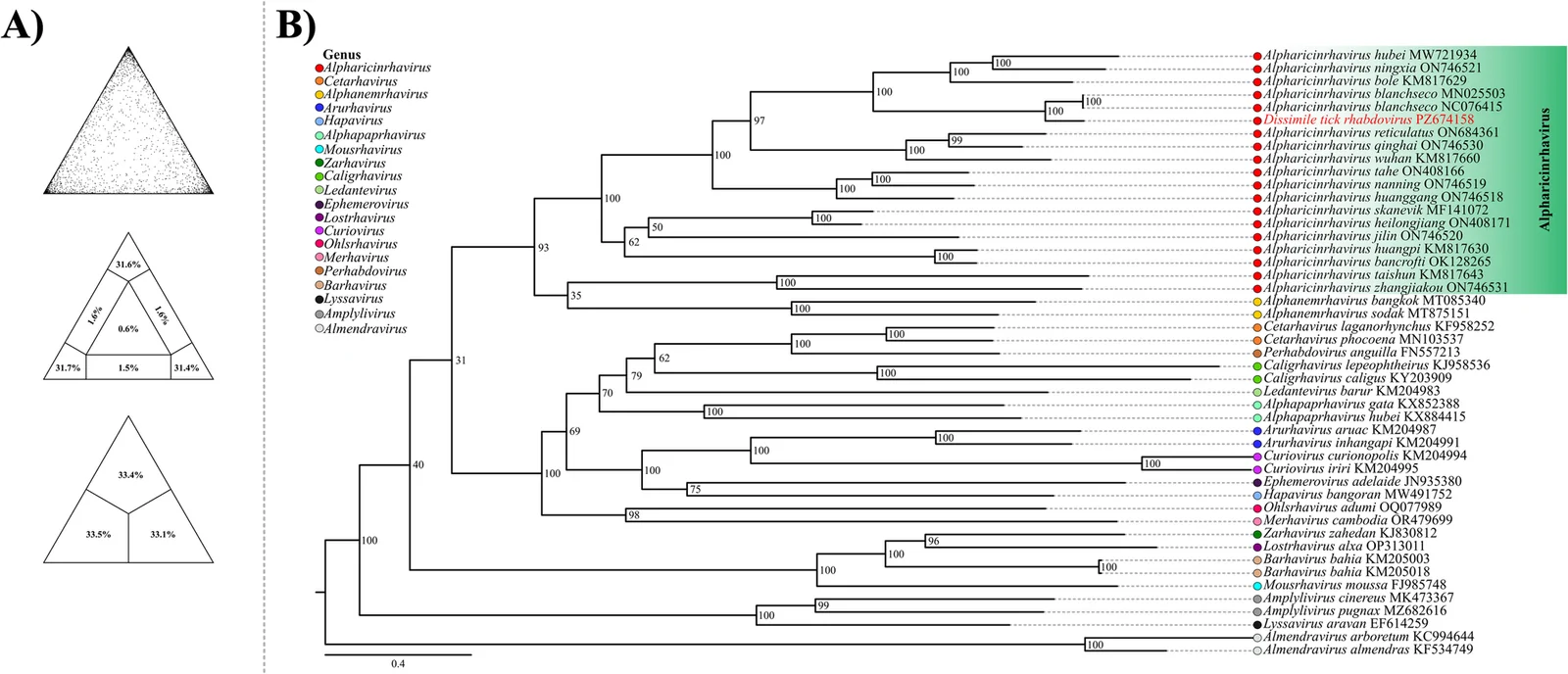
Phylogenetic inferences. **A**) Analysis of the phylogenetic signal of the *L* gene sequence set, used to assess the consistency of the phylogenetic information present in the alignment. **B**) Phylogenetic tree constructed using the *L* gene amino acid substitution model: LG + F+R7. Each genus in the family is represented by a color. The obtained sequence is highlighted in red

When evaluating phylogenetic relationships, the analysis of phylogenetic signal revealed excellent support, with terminal branches showing cumulative values exceeding 90%, indicating high reliability for the phylogenetic inference (Fig. 5A). In the phylogenetic analyses, *Dissimile tick rhabdovirus* PZ674158 clustered within the clade formed by *Alpharicinrhavirus* and *Alphanemrhavirus*; the obtained sequence formed a subclade with the other *Alpharicinrhavirus blanchseco* sequences with a maximum UFB value (100%) (Fig. 5B). Furthermore, a 1,395 bp sequence was detected, showing amino acid identity (75.9%) with the *Alpharicinrhavirus blanchseco* (NC076415) nucleoprotein (Supplementary S3), as well as the presence of a conserved domain related to the *Rhabdoviridae* nucleocapsid (Supplementary S4).

Following the identification of the *L*, *G*, and *N* genes, BLASTx analysis was performed to confirm the identity of the encoded proteins (Table 3). Subsequently, in accordance with the recommendations of the International Committee on Taxonomy of Viruses (ICTV), independent amino acid sequence alignments were conducted for each of the genes (*L*, *G*, and *N*) using a representative set of sequences from the *Rhabdoviridae* family. These alignments enabled the assessment of amino acid identity among the sequences, providing data for the molecular characterization and taxonomic inference of the virus (Table 4).

**Table 3:** Characterization of the *L*, *G*, and *N* genes of the *Dissimile tick rhabdovirus* genomes via identity analysis using Blastx. Shown are the length of each gene (nt), the closest virus identified in GenBank, the corresponding viral protein, amino acid identity (%), coverage (QC, %), and statistical significance value (E-value). QC = Query Cover; P = Polymerase; G = Glycoprotein; N = Nucleoprotein

Virus name (GenBank)	Gene	Length (nt)	Closet virus (GenBank Accession)	Blastx			
				Region	Identity (%)	QC (%)	E-value
*Dissimile tick rhabdovirus* (PZ674158)	L	6,594	*Alpharicinrhavirus blanchseco* (NC076415)	P	84.90	98	0.0
	G	1,560	*Alpharicinrhavirus blanchseco* (NC076415)	G	67.59	96	0.0
	N	1,395	*Alpharicinrhavirus blanchseco* (NC076415)	N	75.66	97	0.0

**Table 4:** Amino acid (AA) and nucleotide (NT) identity of the *L*, *G*, and *N* genes of *Dissimile tick rhabdovirus* following alignment with representative viruses of the family *Rhabdoviridae*. For each gene, the sequence length (nt), the virus showing the highest identity, and the amino acid and nucleotide identity percentages obtained from the alignments are presented. Analyses were performed separately for each gene, in accordance with the recommendations of the International Committee on Taxonomy of Viruses (ICTV), to support the molecular characterization and taxonomic classification of the viruses. P = Polymerase; G = Glycoprotein; NP = Nucleoprotein

Virus name (GenBank Accession)	Gene	Length (nt)	Closet virus (GenBank Accession)	Identity after alignment	
				AA (%)	NT (%)
*Dissimile tick rhabdovirus* (PZ674158)	L	6,594	*Alpharicinrhavirus blanchseco* (NC076415)	84.5	72.6
	G	1,560	*Alpharicinrhavirus blanchseco* (NC076415)	67.4	64.9
	N	1,395	*Alpharicinrhavirus blanchseco* (NC076415)	73.9	69.8

#### Orthomyxoviridae

The *Orthomyxoviridae* family was detected only in *A. dissimile* PZ569664. Five sequences were identified; based on preliminary BLASTx results, these sequences were associated with the amino acid chains of the *PB1*, *PB2*, *PA*, *HA*, and *NP* genes of *Granville quaranjavirus* (Table 2). Furthermore, conserved domains were detected in the *PB1* gene (Fig. 7A).

**Fig. 6:**
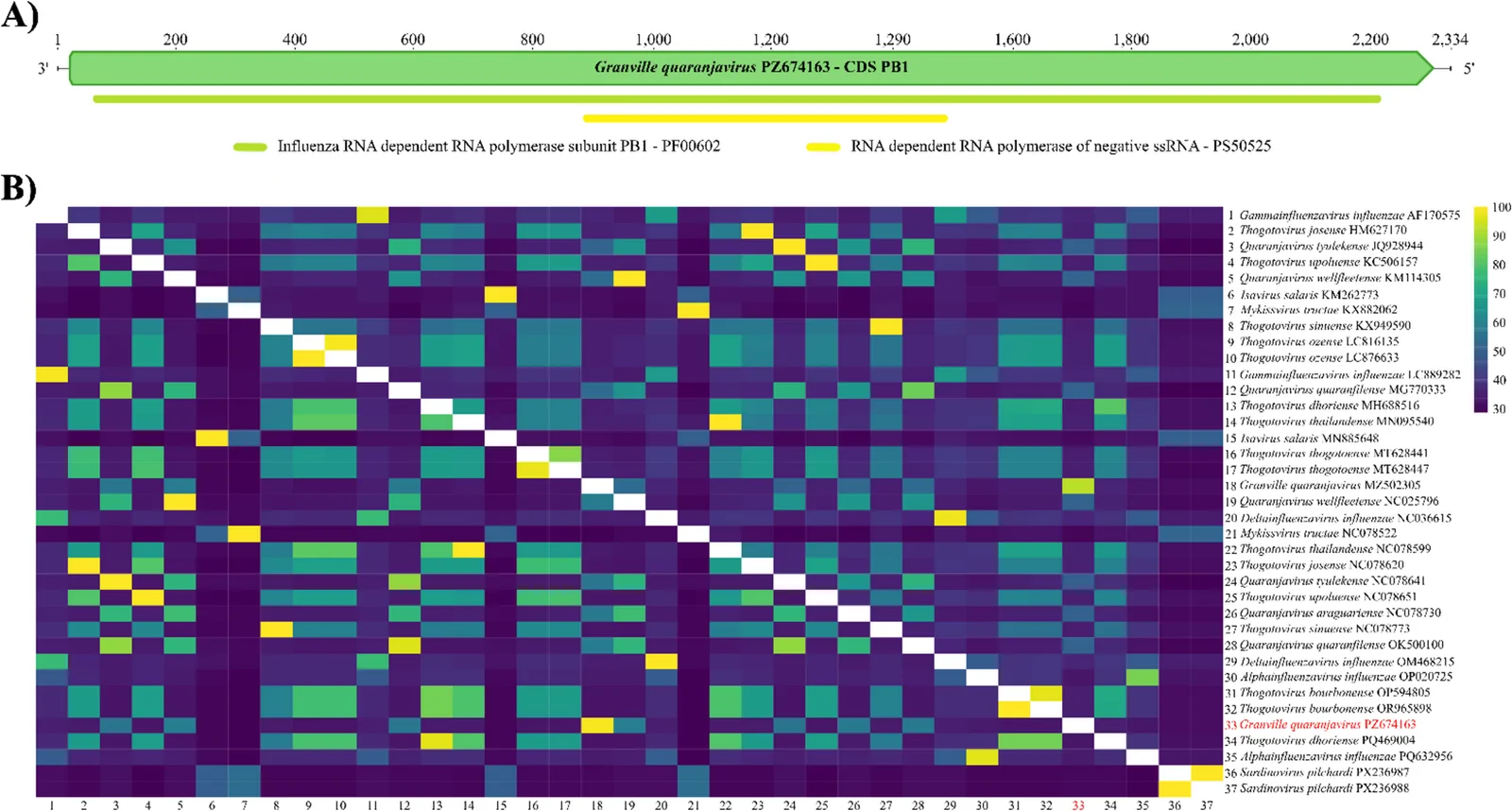
Genomic characterization of *Granville quaranjavirus* PZ674163. **A**) Genomic organization of the *PB1* gene, showing the location of conserved Influenza RNA-dependent RNA polymerase (RdRp) domains (RdRp, PF00602; RdRp, PS50205). **B**) Heatmap of amino acid and nucleotide identity for the *PB1* gene based on pairwise comparisons among representative sequences of the *Orthomyxoviridae* family; the color scale indicates the percentage of identity. The lower and upper triangles show amino acid and nucleotide identity percentages, respectively. The obtained sequence is highlighted in red

**Fig. 7:**
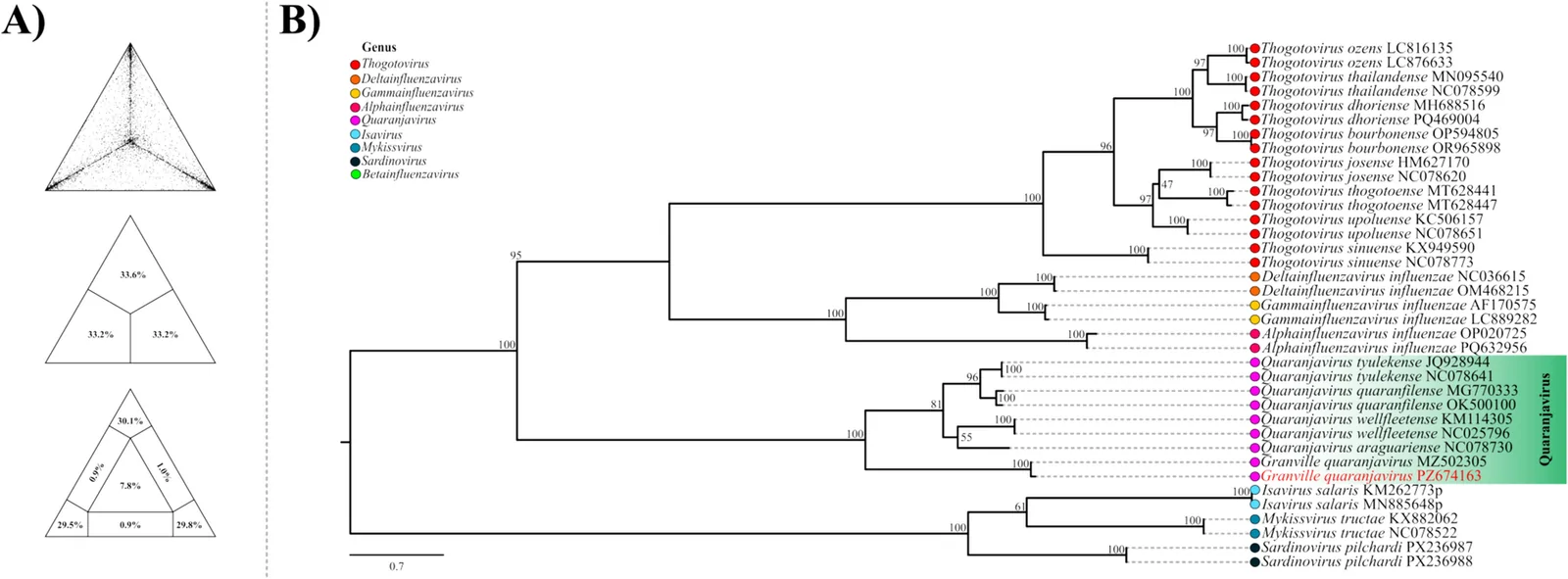
Phylogenetic inferences. **A**) Analysis of the phylogenetic signal of the *PB1* gene sequence set, used to assess the consistency of the phylogenetic information present in the alignment. **B**) Phylogenetic tree constructed using the LG + F+I+G4 amino acid substitution model for the *PB1* gene. Each genus within the family is represented by a color. The virus described in this study is highlighted in red

Amino acid and nucleotide alignments of the identified sequences showed high identity values with *Granville quaranjavirus* sequences (Supplementary S5). The *PB1* gene, in particular, exhibited 98.3% amino acid identity and 92.7% nucleotide identity with *Granville quaranjavirus* (MZ502305) (Fig. 6B; Supplementary S5). Phylogenetic analysis yielded robust support, with terminal branches showing combined values exceeding 89%, indicating high reliability for the phylogenetic inference (Fig. 7A). This result corroborates the phylogenetic analysis of the gene, which formed a subclade with *Granville quaranjavirus* (MZ502305) (UFB = 100%) (Fig. 7B). Similar results were observed in the phylogenetic inferences performed for the *PB2*, *PA*, *HA*, and *NP* genes (Supplementary S6).

#### Nodaviridae

**Fig. 8:**
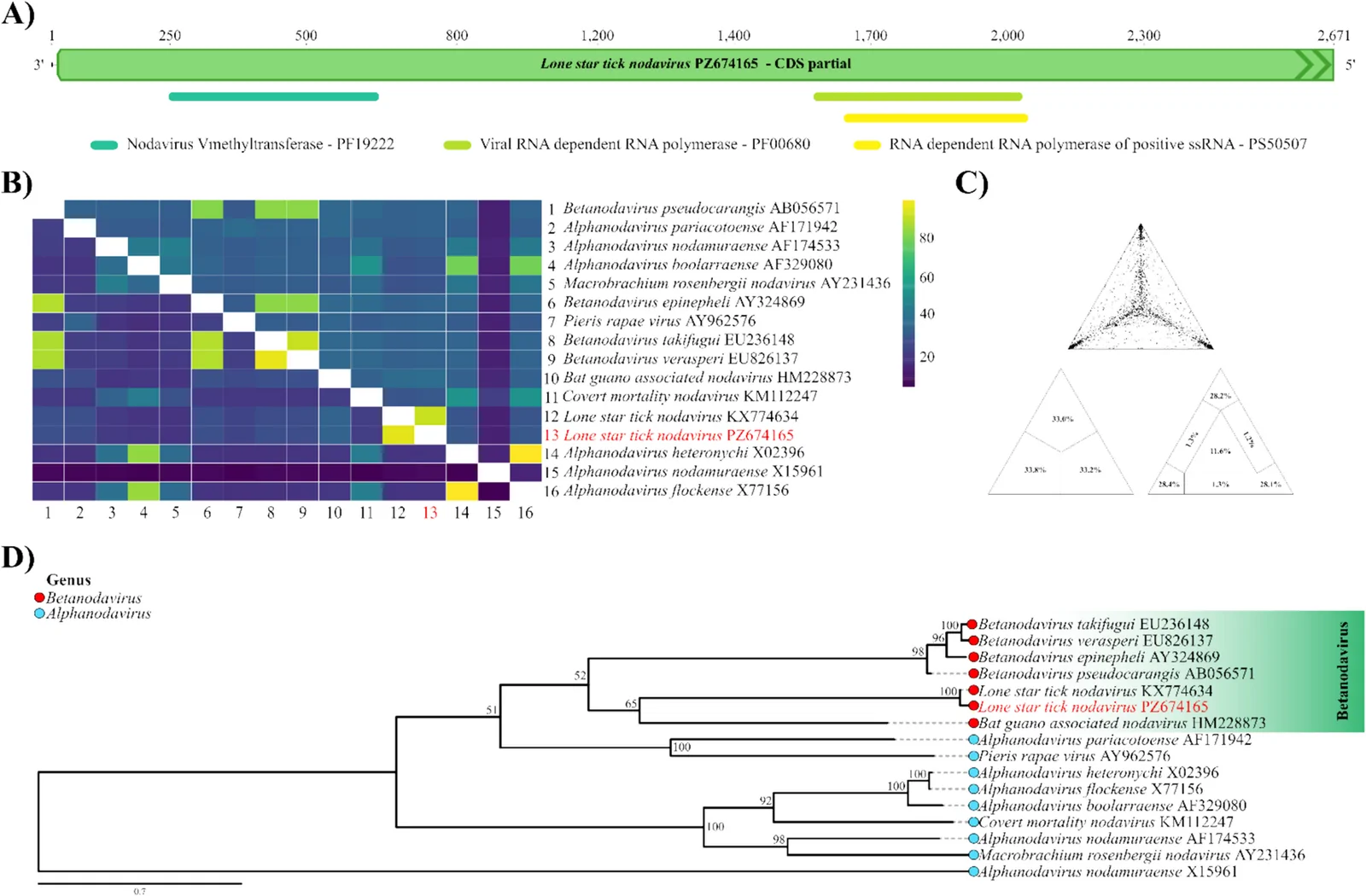
Genomic and phylogenetic characterization of *Lone star tick nodavirus* Z674165. **A**) Lone star tick nodavirus L gene, showing the location of the conserved RNA-dependent RNA polymerase (RdRp) domain (PF00680; PS50507). **B**) Amino acid identity heatmap of the *L* gene based on pairwise comparisons among representative sequences of the *Nodaviridae* family; the color scale indicates the percentage of identity. The lower and upper triangles display amino acid and nucleotide identity percentages, respectively. **C**) Phylogenetic signal analysis of the *L* gene sequence set, used to assess the consistency of the phylogenetic information within the alignment. **D**) Phylogenetic tree constructed using the LG + F+I+G4 amino acid substitution model for the *L* gene. Each genus within the family is represented by a specific color. The virus described in this study is highlighted in red

A 2,671 bp sequence was identified in *A. dissimile* (PZ569664). Blastx analysis matched the polymerase of *Lone star tick nodavirus* (KX774634) with 98.85% identity (Table 2). Although the *Lone star tick nodavirus* PZ674165 sequence detected in the present study is partial, the ORF exhibited conserved domains related to the *Nodaviridae* RdRp (Fig. 8A).

Amino acid and nucleotide identity matrices revealed that the obtained sequence showed similarity percentages of 94.7% and 91.1% with *Lone star tick nodavirus* (KX774634), respectively (Fig. 8B; Supplementary S7). Phylogenetic signal analysis yielded satisfactory support results, with terminal branches showing cumulative values exceeding 84%, indicating the reliability of the phylogenetic inference (Fig. 8C). In the phylogenetic analyses, *Lone star tick nodavirus* PZ674165 clustered with *Lone star tick nodavirus* (KX774634) with 100% UFB support (Fig. 8D).

#### Orthototiviridae

**Fig. 9:**
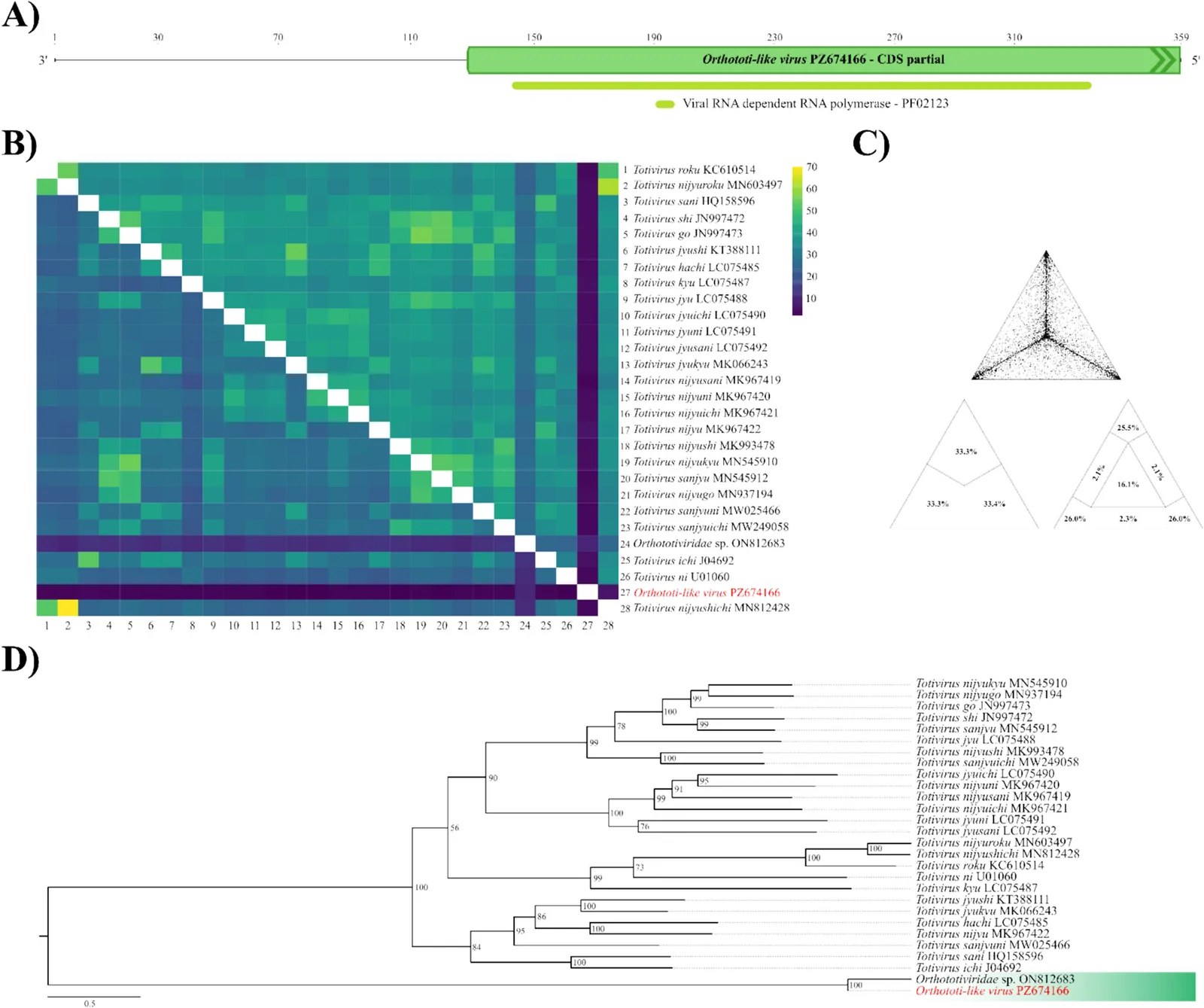
Genomic and phylogenetic characterization of *Orthototi-like virus* 1 PZ674166. **A**) *Orthototi-like virus* 1 *L* gene, showing the location of the conserved RNA-dependent RNA polymerase (RdRp) domain (PF02123). **B**) Amino acid identity heatmap of the *L* gene based on pairwise comparisons among representative sequences of the family *Orthototiviridae*; the color scale indicates the percentage of identity. The lower and upper triangles show amino acid and nucleotide identity percentages, respectively. **C**) Phylogenetic signal analysis of the *L* gene, used to assess the consistency of the phylogenetic information present in the alignment. **D**) Phylogenetic tree constructed using the LG + I+G4 amino acid substitution model for the *L* gene. Each genus within the family is represented by a specific color. The virus described in this study is highlighted in red

A 359 bp sequence was detected, and Blastx results associated the sequence with the family *Orthototiviridae* (Table 2). Although *Orthototi-like virus* PZ674166 presents a partial ORF, a conserved functional domain region related to RdRp was identified (Fig. 9A). The identity matrix showed amino acid and nucleotide identity rates of 90.2% and 77%, respectively, with *Orthototiviridae* sp. (ON812683) (Fig. 9B; Supplementary S8). Phylogenetic signal analysis revealed lower-than-expected support, with the sum of splits showing values below 80%, indicating reduced phylogenetic resolution for this set of sequences. Nevertheless, the phylogenetic tree was used to infer evolutionary relationships among the taxa, also taking branch support values into account (Fig. 8C). In the phylogenetic analyses, *Orthototi-like virus* 1 clustered with *Orthototiviridae* sp. (ON812683), forming a subclade with high support (UFB = 100%) (Fig. 9D).

## Discussion

Ticks are important vectors of infectious agents, such as bacteria and viruses [105]. These infections are frequently reported in vertebrates, resulting in significant impacts on public health, veterinary medicine, and the economic sector [28, 94, 98]. Viral agents, particularly RNA viruses, exhibit high mutation rates due to the low fidelity of RNA polymerases and, in most cases, the absence of repair mechanisms [21]. This high mutation rate can generate strains that are more virulent and capable of causing more severe infections in vertebrates [22, 101]. In the Americas, particularly in North America, infections caused by tick-borne viruses are frequently reported [75, 104]. In contrast, there are few records of infections caused by viruses transmitted by these arthropods in Central and South America [14, 60]. Underreporting is believed to occur in these regions, as the clinical symptoms caused by these pathogens resemble those of various other infections, such as influenza-like illnesses [20, 78], thereby masking the true landscape of diseases caused by tick-borne viruses.

The family *Chuviridae* comprises 16 viral genera characterized by a negative-sense RNA genome of approximately 9 to 12 kb. Furthermore, they are frequently associated with arachnids and other invertebrates in the Americas. This family features between two and four ORFs encoding a glycoprotein, a nucleoprotein, and a large protein containing an RdRp domain [46]. The genus *Mivirus*, in particular, has been primarily associated with ticks of the family Ixodidae [31, 37, 49, 84]. Although members of this genus have largely been discovered via high-throughput sequencing ([31, 84]; GenBank: KU230451, MK026591), the pathogenic potential of *Mivirus* remains unknown, and there are no reports of viral isolation. In the present study, two segments related to the family *Chuviridae* were detected; these clustered within the *Mivirus* genus clade. While the segments show similarities to the nucleoproteins of *Nanning Chuvi tick virus* 2 and *Mivirus changpingense*, the low amino acid identity suggests significant evolutionary divergence. The absence of detectable conserved domains may reflect this divergence, as highly divergent viral proteins often evade recognition by conserved domain databases, particularly when representing lineages that are still poorly sampled [45, 53, 65]. Furthermore, available signature databases remain limited for arthropod viruses identified via metagenomics; thus, the absence of conserved domains does not rule out protein functionality or homology with *Chuviridae* nucleoproteins [10, 47, 83].

The *Rhabdoviridae* family comprises viruses with negative-sense RNA genomes of approximately 10–12 kb [102]. This family consists of 62 genera distributed across four subfamilies. Furthermore, it infects a wide range of hosts, including vertebrates, invertebrates, plants, fungi, and protozoa [2]. The subfamily *Alpharhabdovirinae* infects both vertebrates and invertebrates [91]. Among the members of this subfamily, the genus *Lyssavirus* is the most significant for public and veterinary health, as it includes the rabies virus [29]. *Alpharicinrhavirus blanchseco* (BCOV) was first described in *Amblyomma ovale* specimens collected in Trinidad and Tobago [84]. Additionally, that study involved the first viral isolation, designated as TTP-pool-17. Although there are no reports of infection in vertebrates, BCOV has been classified as a potential zoonotic viral risk [51]. In the present study, a rhabdovirus detected in *A*. *dissimile* collected from a *Boa constrictor* showed a phylogenetic relationship with *Alpharicinrhavirus blanchseco*. Based on the host and sample origin, we named it *Dissimile tick rhabdovirus* (GenBank: PZ674158). However, amino acid identity analyses of the L, N, and G genes (Table 4; Supplementary S5) demonstrated low identity between the virus detected in this study and *Alpharicinrhavirus blanchseco*, indicating considerable genetic divergence and reinforcing that this agent represents a possible new virus within the *Rhabdoviridae* family.

*Orthomyxoviridae* is a family of negative-sense, single-stranded RNA viruses comprising seven genera. The genera *Quaranjavirus* and *Thogotovirus* are of significant interest to public and veterinary health, as they include tick-borne arboviruses [61, 80, 87]. The genus *Quaranjavirus* is one of the most recently recognized genera within the *Orthomyxoviridae* family; it was formally approved by the ICTV in 2013 after phylogenetic studies demonstrated that its members constituted a distinct orthomyxovirus lineage. *Granville quaranjavirus* (GQV) was identified in *A. dissimile* ticks collected from wild animals in Trinidad and Tobago; although it belongs to a genus of medical and veterinary interest, its pathogenicity remains unknown to date [85]. Furthermore, GQV has a geographic distribution restricted to Trinidad and Tobago. In the present study, we report, for the first time in Brazil, the occurrence of Granville quaranjavirus in an *A. dissimile* tick specimen collected from a *Boa constrictor*.

The *Nodaviridae* family comprises two genera: *Alphanodavirus* and *Betanodavirus*. These viruses possess a genome consisting of two positive-sense, single-stranded RNA molecules, RNA1 and RNA2 [82]. Furthermore, they are known to primarily infect fish, insects, and other invertebrates [63, 96]. The first representative of this family (Nodamura virus) was isolated from *Culex tritaeniorhynchus* mosquitoes collected in Nodamura, Japan [90].

Some members of the family *Nodaviridae* are known to be pathogenic to animal hosts. In insects, infection by viruses of the genus Alphanodavirus has been associated with atrophy, paralysis, and mortality. Similarly, in fish, viruses of the genus *Betanodavirus*, including striped jack nervous necrosis virus and red-spotted grouper nervous necrosis virus, can cause neural necrosis or retinopathy and are associated with high mortality, resulting in significant economic losses in aquaculture [41, 63].

Furthermore, the *Nodaviridae* family includes several unclassified viruses; among them, *Lone star tick nodavirus* 1 (KX774634) (LSTN-1) was identified via metagenomics in *Amblyomma americanum* and represents the first member of the *Nodaviridae* family described in ticks [96]. In this study, we report the first occurrence of *Lone star tick nodavirus* (GenBank: PZ674165) in Brazil, detected in *A. dissimile*. Although it is phylogenetically close to arthropod nodaviruses, its ecology, hosts, and zoonotic potential remain unknown.

The genus *Totivirus* comprises viruses with double-stranded RNA (dsRNA) genomes, typically ranging from approximately 4.6 to 7.0 kb in length [103]. Phylogenetic analyses have shown that these viruses cluster with the genera *Leishmaniavirus*, *Giardiavirus*, and *Victorivirus*, which were previously classified within the family *Totiviridae*. However, based on taxonomic revisions conducted by the International Committee on Taxonomy of Viruses (ICTV), members of the former genus *Totivirus* have been reclassified into the family *Orthototiviridae* [86]. Concurrently, the widespread application of high-throughput sequencing (HTS) has significantly expanded knowledge regarding the diversity of these viruses, enabling the discovery of numerous toti-like viruses, primarily associated with plants. These novel viruses have contributed to broadening the taxonomic diversity currently recognized within the family *Orthototiviridae* [1, 13, 86].

With the advancement of genomic approaches, particularly the adoption of high-throughput sequencing (HTS) technologies, an increasing number of viruses belonging to the family *Orthototiviridae* have been identified in ticks of the families Argasidae and Ixodidae (Ni et al., 2023) [42, 48, 73, 95]. Most of these toti-like viruses were discovered exclusively through HTS-based metagenomic studies, and to date, there is no evidence indicating their pathogenic potential for vertebrates.

Although the identification of new viruses in ticks has significantly expanded knowledge regarding the viral diversity associated with these arthropods, a major challenge in high-throughput sequencing (HTS)-based virology remains determining the true host of the detected viruses [17, 68, 97]. This is because many viruses identified through metagenomic approaches may be associated not only with the tick itself but also with symbiotic microorganisms, parasites, or even residues from the blood meal, complicating the determination of their biological specificity and ecological relationships with the host [97] (Ni et al., 2023).

In this context, Insect-Specific Virus (ISVs) serve as an important model for understanding how host specificity can be experimentally demonstrated [9, 23]. Although most ISVs have been described in species of the class Insecta, recent evidence indicates that viral families traditionally considered restricted to these viruses have also been detected in other arthropod groups, suggesting that their host range may be broader than previously recognized [15, 58]. These findings underscore the need to broaden the concept of arthropod-specific viruses by incorporating viral lineages associated with different taxa within this phylum and considering host specificity within a more comprehensive evolutionary context.

Analogous to the concept of insect-specific viruses (ISVs), it may be hypothesized that viruses restricted to arachnid hosts could, in the future, be considered within a broader ecological category of arachnid-associated or arachnid-specific viruses (ASVs). Likewise, viruses detected across different arthropod classes but apparently unable to establish productive infections in vertebrates could potentially be considered within the broader concept of arthropod-specific viruses. However, these concepts should currently be regarded as theoretical possibilities rather than established biological or taxonomic categories. Sequence detection alone is insufficient to determine host specificity, as viral sequences may originate from transient associations, including residual nucleic acids from a blood meal, environmental contamination, or other components of the arthropod-associated microbiota.

Establishing host specificity would require systematic and reproducible experimental and ecological evidence, including, where applicable: (i) isolation and demonstration of efficient viral replication in cells derived from the putative arthropod host; (ii) assessment of replication in vertebrate cells; (iii) experimental evidence regarding the ability or inability to establish productive infection in vertebrate models; (iv) ecological evidence supporting persistent maintenance within natural host populations; and (v) phylogenetic evidence demonstrating consistent associations between viral lineages and particular host groups. Therefore, the viruses identified in the present study should not be considered ASVs or other host-specific viruses based solely on their detection in a single tick specimen. Rather, our findings constitute preliminary evidence of viral diversity associated with ticks in Brazil and provide a basis for future investigations aimed at determining host range, replication competence, ecological persistence, and potential host specificity.

Although currently available evidence does not yet al.low for their classification as Arachnid-Specific Virus or, more broadly, as Arthropod-Specific Virus, the genomic characterization performed in this study significantly expands knowledge regarding the tick-associated virosphere and represents an important advance in understanding the viral diversity of these ectoparasites in Brazil. Furthermore, these findings establish a foundation for future investigations aimed at elucidating their natural hosts, host specificity, transmission cycles, and biology, as well as their potential placement within a classification system based on host taxonomic restriction.

Identifying new viruses with pathogenic potential is crucial for understanding the role of ticks as vectors of agents capable of emerging and causing outbreaks or epidemics, as well as for assessing the risks associated with their circulation and spread [108]. Furthermore, knowledge of the viral diversity of these arthropods supports the development of more sensitive diagnostic and epidemiological surveillance tools, helping to elucidate the etiology of febrile syndromes of unknown origin [17, 56]. In this context, studies characterizing the tick RNA virome are increasingly relevant given global climate change, as environmental alterations can modify the geographic distribution, abundance, and population dynamics of these ectoparasites, directly influencing the transmission of the viruses and other infectious agents they carry [24, 71, 105]. Thus, expanding knowledge regarding tick-associated viral diversity is an essential component of genomic surveillance strategies and preparedness for the emergence of new pathogens.

Studies based on high-throughput sequencing (HTS) are fundamental to expanding our understanding of viral ecology, virus-vector interactions, and the mechanisms governing virus circulation and co-circulation in natural tick populations [36, 64, 92]. Furthermore, the results obtained in this study highlight the underrepresentation of viral diversity associated with Brazilian ticks in international reference databases, underscoring the need to intensify metagenomic surveillance and virological characterization efforts in Brazil. In this context, the genomic data presented here expand knowledge regarding the tick virosphere in the country and provide a baseline for future investigations into the evolution, ecology, host specificity, and zoonotic potential of viruses associated with these arthropods.

The present study was based on a single *A. dissimile* specimen (*n* = 1), which represents an important limitation for extrapolating the observed viral diversity to broader ecological or epidemiological patterns. Nevertheless, the analysis of an individual tick was intentionally adopted to preserve a direct association between the molecularly identified tick specimen and its associated viral sequences. This approach is particularly relevant for *Amblyomma*, a genus that includes closely related species and species complexes, for which pooling specimens could compromise species-level attribution of detected viruses. Further studies involving larger numbers of individually identified ticks from different populations, hosts, and geographic locations will be necessary to determine the prevalence, distribution, host associations, and ecological significance of the viruses identified here.

## Conclusion

This study provides the first comprehensive characterization of the RNA virome associated with *Amblyomma dissimile* in the Amazon, particularly in the Brazilian Amazon. Key findings include the identification of *Dissimile tick rhabdovirus*, a potential new member of the *Rhabdoviridae* family described here for the first time, with no prior records in the scientific literature. Additionally, segments of *Granville quaranjavirus* were detected for the first time in Brazil, expanding the known geographic distribution of this virus. Various segments and fragments of unclassified viruses were also identified, highlighting the high viral diversity associated with this tick species. Phylogenetic analyses revealed consistent topologies with strongly supported clades, corroborating the evolutionary placement of the identified viruses and indicating the existence of viral lineages that remain poorly characterized. Together, these results highlight the underrepresentation of viral diversity associated with Brazilian ticks in reference databases, reinforcing the need to expand metagenomic surveillance and virological characterization efforts in the country, especially given the potential impacts of climate change on tick distribution and virus circulation dynamics. Given that the pathogenicity of most tick-associated viruses remains unknown, future studies should prioritize investigating their host specificity, transmission capacity, biology, vector competence, and pathogenic potential. Advancing this knowledge will be crucial for understanding the risk of emerging tick-borne viruses and for informing the development of genomic surveillance, prevention, and control strategies for infectious diseases of importance to human, animal, and environmental health.

## Supplementary Information

Below is the link to the electronic supplementary material.

Supplementary File 1 (XLSX 36.5 KB)

Supplementary File 2 (XLSX 14.8 KB)

Supplementary File 3 (XLSX 20.6 KB)

Supplementary File 4 (XLSX 25.1 KB)

Supplementary File 5 (XLSX 48.1 KB)

Supplementary File 6 (PDF 80.8 KB)

Supplementary File 7 (PDF 80.2 KB)

Supplementary File 8 (XLSX 94.9 KB)

## Data Availability

All data generated during this study are available as tables and Figure s included in this published article and its Supplementary Information files. The accession numbers in the GenBank database for the viral genomes detected in this study are PZ674163 (Granville quaranjavirus), PZ674164 (Granville quaranjavirus), PZ674162 (Granville quaranjavirus), PZ674160 (Granville quaranjavirus), PZ674161 (Granville quaranjavirus), PZ674156 (Dissimile tick chuvirus 1), PZ674157 (Dissimile tick chuvirus 2), PZ674158 (Dissimile tick rhabdovirus), PZ674159 (Dissimile tick rhabdovirus), PZ674165 (Lone star tick nodavirus) e PZ674166 (Orthototi-like virus).
