# Supplementary material for: Detection, characterization, and evolutionary inferences regarding a novel virus identified in Amblyomma dissimile Koch, 1844 (Acari: Ixodidae) from the Amazon, Pará, Brazil
Source: Arch Virol. 2026 Oct 23;171(11):304. doi: 10.1007/s00705-026-06764-5 (PMC13694466; doi:10.1007/s00705-026-06764-5)
Supplement: Supplementary file 1 [file 705_2026_6764_MOESM6_ESM.pdf]

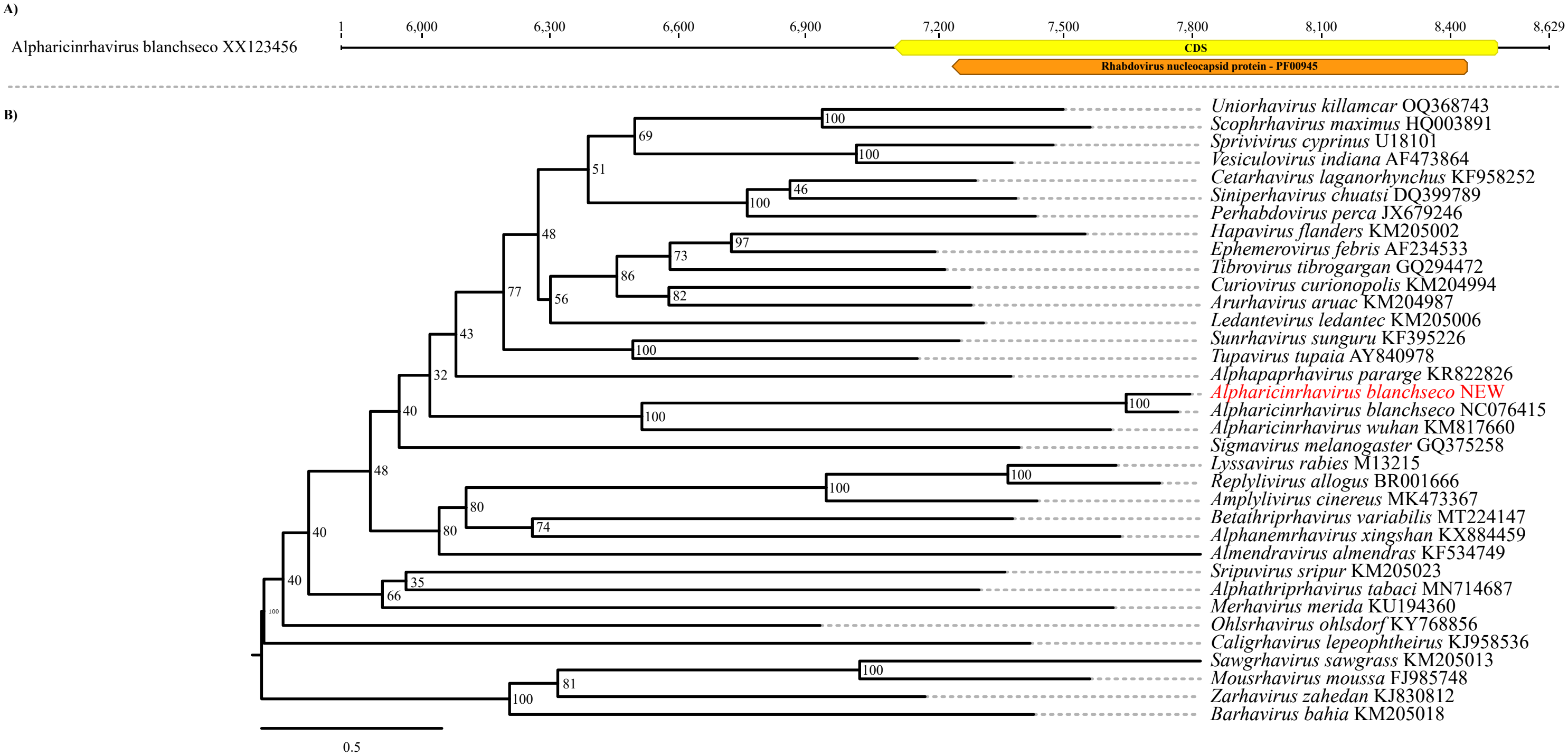

Figure S2. Phylogenetic inferences for the detected sequence related to the *Rhabdoviridae* family, constructed using the best amino acid substitution model: LG+R5. The obtained sequence is highlighted in red.
