# Supplementary material for: Detection, characterization, and evolutionary inferences regarding a novel virus identified in Amblyomma dissimile Koch, 1844 (Acari: Ixodidae) from the Amazon, Pará, Brazil
Source: Arch Virol. 2026 Oct 23;171(11):304. doi: 10.1007/s00705-026-06764-5 (PMC13694466; doi:10.1007/s00705-026-06764-5)
Supplement: Supplementary file 2 [file 705_2026_6764_MOESM7_ESM.pdf]

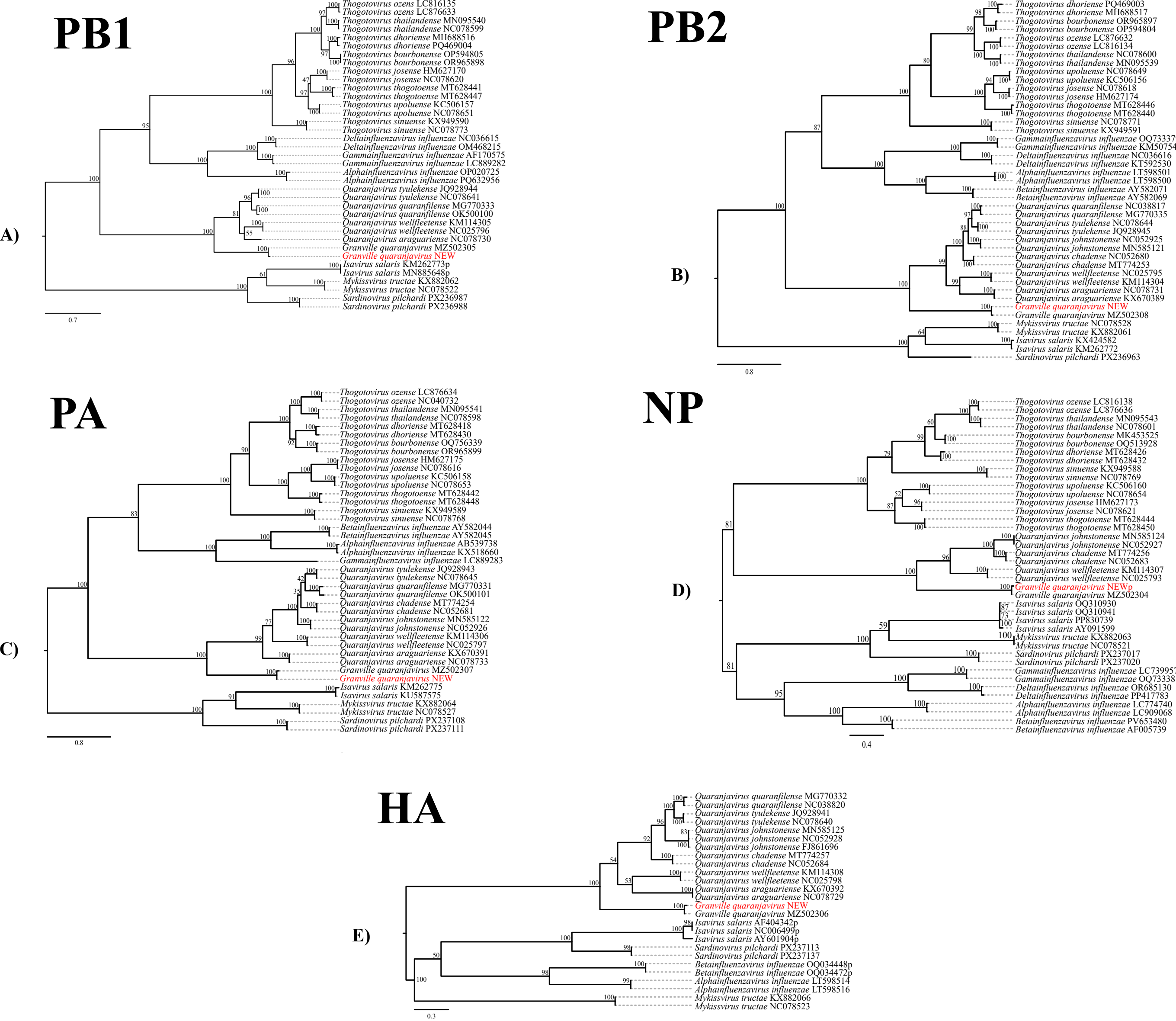

Figure S1 - Phylogenetic inferences of the detected sequences related to the *Orthomyxoviridae* family, constructed with the best amino acid substitution model of CDS: A) LG+F+I+G4 (PB1); B) LG+F+I+G4 (PB2); C) LG+F+I+G4 (PA); D) LG+F+I+G4 (NP) and E) LG+F+I+G4 (HA). The obtained sequences are highlighted in red.
